# Celebrating 120 Years of Butantan Institute Contributions for Toxinology

**DOI:** 10.3390/toxins14020076

**Published:** 2022-01-21

**Authors:** Ana M. Moura-da-Silva

**Affiliations:** Laboratório de Imunopatologia, Instituto Butantan, Av. Vital Brasil, 1500, São Paulo 05503-900, SP, Brazil; ana.moura@butantan.gov.br

A hundred and twenty years ago, the Butantan Institute was founded by the Brazilian physician and scientist Vital Brazil, combining, in the same institution, medical research, and the transfer of results to society in the form of health products. Its foundation was a reaction to the outbreak of bubonic plague in the city of Santos, São Paulo State of Brazil, but the Institution soon also showed its specialization in the study of venomous animals, their venoms, and the production of antivenoms. More than a century after its foundation, the Institute maintains its tradition and initial mission regarding important contributions for collective health. Today, Butantan is an outstanding biomedical research center, which integrates basic research, technological development, the production of immunobiological, and scientific divulgation, seeking the permanent updating and integration of its resources. Butantan is internationally known for its research on venomous animals, and houses one of the largest collections of snakes in the world. Butantan scientists have published countless studies on the characterization of the composition of venoms, the mechanisms of action of their toxins, and the use of toxins as lead molecules for the development of new drugs. The Butantan Institute also operates the “Hospital Vital Brazil”, which is specialized in accidents involving venomous animals. The dissemination of scientific knowledge occurs at different levels and media; the institution is a pioneer in the training of graduate students in the field of Toxinology for MSc and PhD degrees.

This Special Issue of *Toxins* celebrates the 120th anniversary of the Butantan Institute, in recognition of their contribution to international Toxinology, highlighting the current production by house scientists and collaborators from other institutions. We selected 19 original articles and 4 reviews approaching several points of Toxinology. The venom of snakes was approached in different aspects as the structural and functional variability in the composition of venoms from individual snakes [1], the mechanisms of action of whole venoms [2,3], the mechanisms of action of individual components as crotoxin [4], phospholipases A2 [5], metalloproteinases [6,7,8], and the oral immunity induced by whole venoms and their components [9]. Venoms from other animals are also reported; the venoms of centipedes [10], scorpions [11,12], fishes [13,14], and caterpillars [15], including microbial toxins [16]. The great potential for molecules derived from animal venoms as drug leads was also reviewed [17], and the antimicrobial [18], anticoagulant [19,20], and analgesic [21,22] effects have been highlighted in original studies reported here.

In conclusion, this is a small recognition of the institute’s contribution to the field of Toxinology that demonstrates its continued and relevant role in this field. I hope you enjoy reading this, and congratulations to the Butantan Institute, scientists, and collaborators, on their 120th anniversary.
